# Gleanings

**Published:** 1898-04

**Authors:** 


### GLEANINGS.

Dr. A. W. Bitting reports 80 per cent. of recoveries in ten cases of actinomycosis treated with a drachm to a drachm and a half of iodide of potassium daily for two weeks.

Crude petroleum is recommended by several large hog-breeders as an excellent exterminator of lice upon hogs. Its use internally as a laxative is also recommended. Its value as a food-product among swine is being discussed.

Jas. M. Hough, of South Dakota, reports the following as his experience in feeding millet to horses: That they grow fat as hogs; of some three mares in foal, one aborted and two had colts so weak-jointed that they never amounted to anything; the fattened horses were weak, and unable to do any hard work, and some of them died while under the usual farm-work. These anmials sometimes require six months to get them in condition after a winter's feeding on millet. Sheep, cows, hogs, turkeys, and chickens do well on it. The latter increase in egg-producing qualities. Native bronchos seem less susceptible to ulterior influences from its use, but heavy horses become physical wrecks under its feeding.

The prosecution in the Luetgert case at Chicago, in the first trial, laughed at the idea of calling "horse doctors" as experts on bones; but in the words of the Chicago *Press,* "the laughing was all done before they testified."

